# Reclassification of variants of tumor suppressor genes based on Sanger RNA sequencing without NMD inhibition

**DOI:** 10.3389/fgene.2023.1283611

**Published:** 2023-10-12

**Authors:** Changhee Ha, Ja-Hyun Jang, Young-gon Kim, Jong-Won Kim

**Affiliations:** Department of Laboratory Medicine and Genetics, Samsung Medical Center, Sungkyunkwan University School of Medicine, Seoul, Republic of Korea

**Keywords:** tumor suppressor gene, Sanger RNA sequencing, aberrant splicing, nonsense-mediated mRNA decay, NMD

## Abstract

**Introduction:** RNA sequence analysis can be effectively used to identify aberrant splicing, and tumor suppressor genes are adequate targets considering their loss-of-function mechanisms. Sanger sequencing is the simplest method for RNA sequence analysis; however, because of its insufficient sensitivity in cases with nonsense-mediated mRNA decay (NMD), the use of cultured specimens with NMD inhibition has been recommended, hindering its wide adoption.

**Method:** The results of Sanger sequencing of peripheral blood RNA without NMD inhibition performed on potential splicing variants of tumor suppressor genes were retrospectively reviewed. For negative cases, in which no change was identified in the transcript, the possibility of false negativity caused by NMD was assessed through a review of the up-to-date literature.

**Results:** Eleven potential splice variants of various tumor suppressor genes were reviewed. Six variants were classified as pathogenic or likely pathogenic based on the nullifying effect identified by Sanger RNA sequencing. Four variants remained as variants of uncertain significance because of identified in-frame changes or normal expression of both alleles. The result of one variant was suspected to be a false negative caused by NMD after reviewing a recent study that reported the same variant as causing a nullifying effect on the affected transcript.

**Conclusion:** Although RNA changes found in the majority of cases were expected to undergo NMD by canonical rules, most cases (10/11) were interpretable by Sanger RNA sequencing without NMD inhibition due to incomplete NMD efficiency or allele-specific expression despite highly efficient NMD.

## Introduction

RNA sequence analysis is a simple yet powerful tool for pathogenicity classification of germline genetic variants. In the context of germline variant interpretation, the role of RNA sequence analysis is to identify aberrant splicing that leads to the loss of function of an allele via in-frame loss of a critical part of a protein or frameshifting changes. The mRNAs produced by frameshift variants are prone to nonsense-mediated mRNA decay (NMD), a physiological surveillance mechanism (Richards et al., 2015; Anna and Monika, 2018; Tahiliani et al., 2020) that prevents the production of potentially toxic truncated proteins. In RNA sequence analysis, the possibility of a reduction in the amount of mRNA by NMD, even to a level below the limit of detection (LOD, the lowest quantity that can be detected by a method), should be taken into account.

When a specimen is available, the simplest and most effective method for RNA sequence analysis is the Sanger sequencing of cDNA after reverse transcription PCR (RT-PCR) (Anna and Monika, 2018). Sanger RNA sequencing can be performed in most clinical laboratories that perform Sanger DNA sequencing. One limitation of Sanger sequencing in the analysis of RNA is the LOD, which is approximately 15% (Tsiatis et al., 2010), because variant transcripts may exist in a proportion below this level in the case of highly efficient NMD. To overcome the effects of NMD, more sophisticated methods, such as mini-gene assays or cell cultures with NMD inhibition, are required (Anna and Monika, 2018). However, most routine clinical laboratories cannot afford these methods.

It is widely accepted that the efficiency of NMD is highly variable, depending on the position of premature termination codons (PTC). Most well-known factor of NMD efficiency is defined by the canonical rules, namely, the last exon rule and the 50 nt rule. By these two rules, PTCs in the last exon and those in last 50 nt of the penultimate exon are expected to escape NMD, respectively. However, it turned out that there are many other factors affecting NMD efficiency including random variance (Lindeboom et al., 2016; Lindeboom et al., 2019; Supek et al., 2021). According to these reports, the NMD efficiency is often far from 100%, even if the PTC is in the region predicted to undergo NMD by canonical rules. Theoretically, variant transcripts that undergo NMD can be detected by simple Sanger RNA sequencing if the amount of remnant transcripts is greater than the LOD. In a recent study, it was suggested that NMD inhibitor treatment should be used as a second investigation when the initial investigation without NMD inhibition failed to detect positive findings, considering cost-effectiveness (Bournazos et al., 2022).

Tumor suppressor genes are the most widely sequenced genes in the clinical environment and are appropriate targets for RNA sequence analysis because of their loss-of-function disease mechanisms and blood expression (Landrith et al., 2020; Lee et al., 2021; Gu et al., 2022; Na et al., 2022). In addition, the widespread adoption of state-of-the-art *in silico* tools for aberrant splicing prediction, such as SpliceAI (Jaganathan et al., 2019), has increased the need to confirm the consequences of pre-mRNA splicing. However, the fraction of variants whose experimental results can be found in the literature is small, making each laboratory responsible for confirmation. More active sharing of RNA sequence analysis results can help laboratories encountering the same variants.

In this study, the results of Sanger RNA sequencing without NMD inhibition performed on potential splicing variants of tumor suppressor genes were retrospectively reviewed. The interpretation procedure for RNA sequencing of each case, under the effect of NMD, and the consequent pathogenicity classification are specifically described.

## Methods

### Retrospective review of Sanger RNA sequencing results

We retrospectively reviewed patients who underwent Sanger RNA sequencing of peripheral blood from January 2021 to July 2023 at the Samsung Medical Center (SMC) for potential splicing variants found in the DNA sequencing of tumor suppressor genes. For apparently negative cases, in which no change was identified in the transcript, a review of up-to-date literature was performed to determine the recent experimental results for the same variant. This study was performed in accordance with the Declaration of Helsinki and approved by the Institutional Review Board of SMC, Seoul, Korea (approval number 2023-06-090). The requirement for informed consent was waived due to the retrospective nature of the study, which was approved by the institutional review of IRB.

### Sanger RNA sequencing

RT-PCR and cDNA sequencing were performed to investigate altered splicing. Peripheral blood samples were drawn into vacuum tubes containing ethylenediaminetetraacetic acid and RNA was extracted from the leukocytes of patients and healthy controls. RNA was extracted by the TRIzol methods and 1 µg of RNA was reverse transcribed into cDNA using the random hexamer RT primer and RevertAid First Strand cDNA Synthesis Kit (Thermo Fisher Scientific, Waltham, MA, United States). cDNA amplification was performed using a GeneAmp PCR System 9700 Thermal Cycler (Applied Biosystems, Foster City, CA, United States) with target-specific in-house primers (Supplementary Table S1). The size of the bands was estimated by electrophoresis. Sanger RNA sequencing was performed using purified templates. Cyclic sequencing was performed using the BigDye Terminator v3.1 Cycle Sequencing Kit (Applied Biosystems), and sequence traces were obtained on an ABI 3730xl DNA Analyzer (Applied Biosystems). Sequence variations were detected using the Sequencher software version 5.4 (Gene Codes, Ann Arbor, MI, United States). Electrophoresis and sequencing results for each patient were compared with those for healthy controls. Peaks having a height below 15% than that of the main sequence peaks were considered noise.

## Results

**FIGURE 1:**
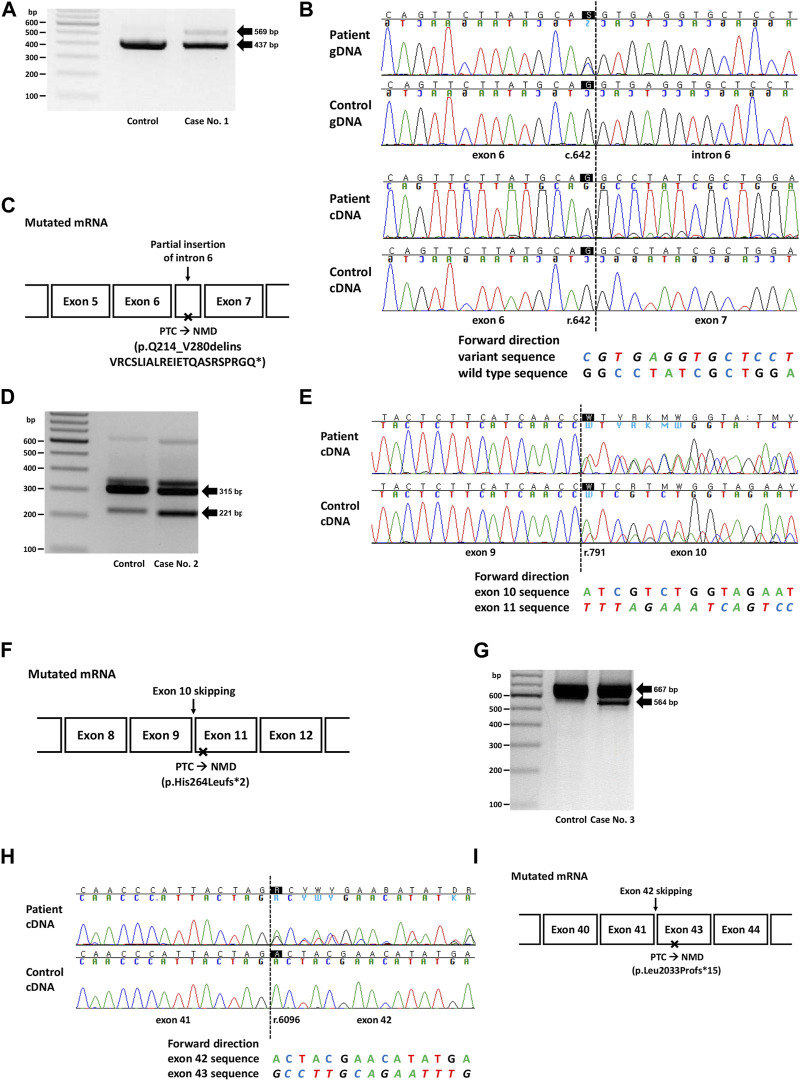
Genetic study results of cases No. 1 (NM_003000.3(SDHB):c.642G>C), No. 2 (NM_000249.4(MLH1):c.791A>T), and No. 3 (NM_000051.4(ATM):c.6096-14A>G). **(A)** RT-PCR electrophoresis results showing a longer transcript produced from the mutant allele in case no. 1. **(B)** Electropherogram of genomic DNA (gDNA) and complementary DNA (cDNA) from case No. 1. The heterozygous SNV (c.642G>C) observed in the DNA sequence was absent in the RNA sequences, suggesting allele-specific expression. The insertion of intron 6 (c.642 + 1_642 + 132ins) was identified as peaks with a lower height in the electropherogram. **(C)** Diagram of the mutated mRNA of case No. 1, with relative position of premature termination codon. **(D)** RT-PCR results showing increased expression of aberrant transcripts in Case No. 2. **(E)** cDNA sequencing results showing skipping of exon 10, with a difference in peak height between case No. 2 (50%) and the control (<50%). **(F)** Diagram of the mutated mRNA of case No. 2, with relative position of premature termination codon. **(G)** RT-PCR electrophoresis results for case No. 3 showing shorter transcripts produced from the mutant allele. **(H)** cDNA sequencing results showing skipping of exon 42 in case No. 3. **(I)** Diagram of the mutated mRNA of case No. 3, with relative position of premature termination codon. **(C, F, I)** Box sizes are not proportional to the length of exon and intron.

**FIGURE 2:**
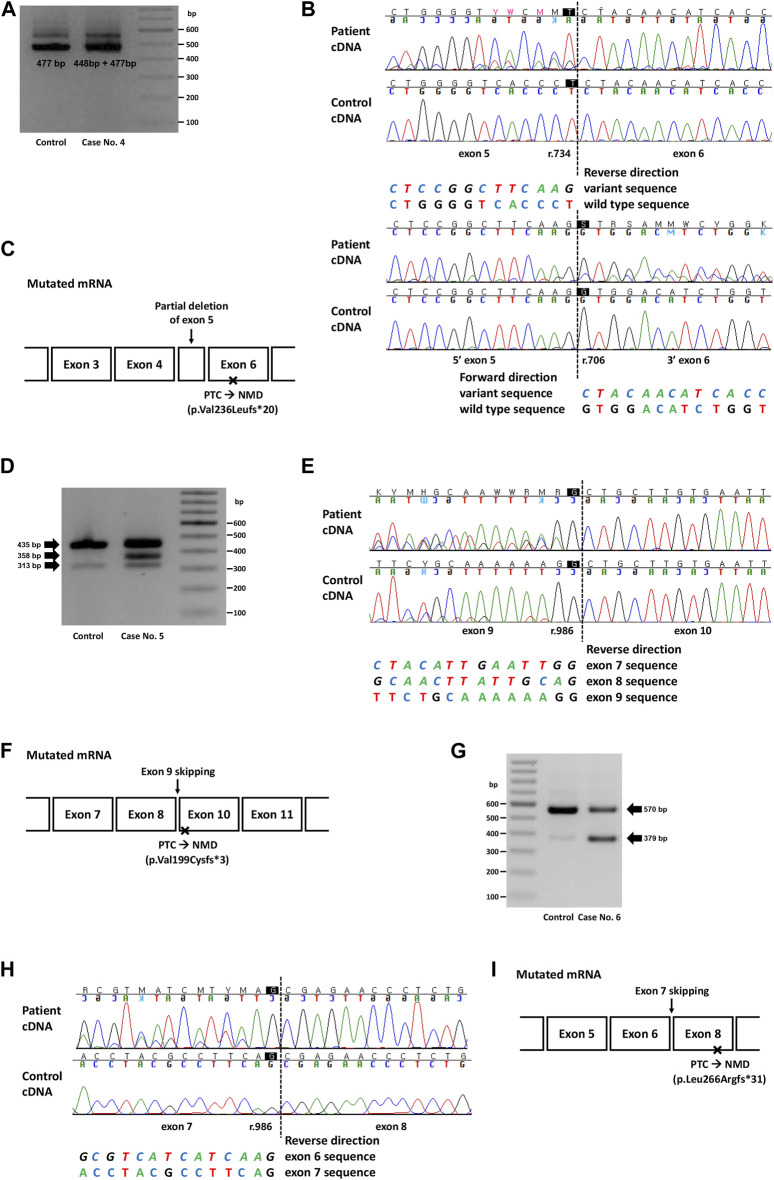
Genetic study result of cases No. 4 (NM_000455.5(STK11):c.734 + 5G>A), No. 5 (NM_007294.4(BRCA1):c.670 + 1G>A) and No. 6 (NM_003073.5(SMARCB1):c.986 + 1_986 + 10delinsTTGGGTTAA). **(A)** RT-PCR electrophoresis result showing shorter transcript produced from mutant allele in case No. 4. **(B)** cDNA sequencing result showing partial deletion of exon 5 (r.706_734del) in case No. 4. **(C)** Diagram of the mutated mRNA of case No. 4, with relative position of premature termination codon. **(D)** RT-PCR electrophoresis result of case No. 5 showing aberrant transcripts produced from mutant allele. **(E)** cDNA sequencing result of case No. 5 and control. In both patient and control, naturally occurring exon 8 and 9 skipping was observed as minor variant peaks. Additional exon 9 skipping was observed only in case No. 5 (major variant peaks). **(F)** Diagram of the mutated mRNA of case No. 5, with relative position of premature termination codon. **(G)** RT-PCR electrophoresis result of case No. 6 showing short aberrant transcript produced from mutant allele. **(H)** cDNA sequencing result showing skipping of exon 7 in case No. 6. **(I)** Diagram of the mutated mRNA of case No. 6, with relative position of premature termination codon. **(C, F, I)** Box sizes are not proportional to the length of exon and intron.

During the study period, Sanger RNA sequencing was performed on 11 patients to identify the splicing effects of potential splicing variants. The RNA sequencing results and clinical information of the 11 patients are described in Table 1. Nine variants were predicted to cause aberrant splicing by SpliceAI (delta score >0.2) and the remaining two had a delta score of 0.17. Eight variants were intronic and three were exonic (two missense and one synonymous). The variants were mostly single-nucleotide variants (SNVs) with one insertion/deletion (INDEL) variant. These variants have been observed in various tumor suppressor genes in patients with diverse malignancies. Various RNA consequences were observed, including the loss of one allele (Case No. 1), exon skipping that led to a frameshift (Cases No. 2, 3, 4, 5, and 6), in-frame insertion of intronic sequences (Cases No. 7, 8, and 9), normal expression of both alleles (Case No. 10), and presumably false-negative results (Case No. 11). Six out of the 11 variants could be classified as pathogenic variants (PV) or likely pathogenic variants (LPV) based on Sanger RNA sequencing results, and four remained as variants of uncertain significance (VUS). The results from family members and the list of patients with the same variants are listed in Supplementary Table S2. Diagrams of each variant along with last exon-exon junction were illustrated in the Figures 1, 2 and Supplementary Figures S3, S4.

**TABLE 1:** RNA study results of 11 patients and their clinical information.

Case no.	Sex/age	Diagnosis	Variant	SpliceAI delta score	gnomAD (v2.1.1)	RNA sequencing result	ACMG classification^a^ (post-RNA study)	Clinvar evidence (N)^b^
1	M/27	Paraganglioma	NM_003000.3(SDHB): c.642G>C, p.(Gln214His)	0.90	Absent	Loss-of-heterozygosity due to NMD	LPV (PVS1, PM2)	VUS (4)
2	M/44	Colon cancer	NM_000249.4(MLH1): c.791A>T, p.(His264Leu)	0.17	Absent	Exon 10 skipping (r.791_884del)	LPV (PVS1, PM2)	VUS (2)
3	F/37	Breast cancer	NM_000051.4(ATM): c.6096-14A>G, p.(?)	0.84	0.0036%	Exon 42 skipping (r.6096_6198del)	LPV (PVS1, PM2)	LBV (5)
4	M/25	Peutz-Jeghers syndrome	NM_000455.5(STK11): c.734 + 5G>A, p.(?)	0.47	Absent	Exon 5 partial deletion (r.706_734del)	PV (PVS1, PM2, PP1_Moderate)	PV (1)
5	F/63	N/A	NM_007294.4(BRCA1): c.670 + 1G>A, p.(?)	0.79	Absent	Exon 9 skipping (r.594_670del)	LPV (PVS1, PM2)	VUS (1)
6	M/22 m	ATRT	NM_003073.5(SMARCB1): c.986 + 1_986 + 10delinsTTGGGTTAA, p.(?)	0.49	Absent	Exon 7 skipping (r.796_986del)	LPV (PVS1, PM2)	No report
7	F/68	Breast cancer	NM_007294.4(BRCA1): c.4186-11C>A, p.(?)	0.99	Absent	r.4185_4186insuuuuugaag (c.4186-9_4186-1ins)	VUS (PM2, PM4)	VUS (1)
8	F/63	Ovarian cancer	NM_007294.4(BRCA1): c.5407-11T>A, p.(?)	1.00	Absent	r.5406_5407insgggauccag (c.5407-9_5407-1ins)	VUS (PM2, PM4)	Not provided (1)
9	F/59	Breast cancer	NM_000059.4(BRCA2): c.8755-19A>G, p.(?)	0.97	Absent	r.8754_8755insuaucuuaaauggucacag (c.8755-18_8755-1ins)	VUS (PM2, PM4)	VUS (4); LBV (4)
10	M/15	Mixed germ cell tumor	NM_002439.5(MSH3): c.2433A>G, p.(Leu811 = )	0.17	Absent	No ASE	VUS (PM2, PP3)	LBV (2)
11	F/43	Endometrial cancer	NM_000251.3(MSH2): c.2635-24A>G, p.(?)	0.69	Absent	Indeterminate	N/A	PV/LPV (5)

^a^ Described as ACMG, classification (evidence).

^b^ N indicates number of submitters, Clinvar accessed 12 August 2023.

N/A, not available; ATRT, atypical teratoid rhabdoid tumor; NMD, nonsense-mediated mRNA, decay; ASE, allele-specific expression; LPV, likely pathogenic variant; PV, pathogenic variant; VUS, variant of uncertain significance; N/A, not available.

Case No. 1 was a 27-year old male patient with paraganglioma. A missense variant in *SDHB* (NM_003000.3:c.642G>C, p.Gln214His) was detected by targeted panel sequencing of pheochromocytoma/paraganglioma-related genes. A retrospective review of medical records revealed that this variant was repeatedly detected in many patients in SMC who had paraganglioma and/or pheochromocytoma (Supplementary Table S2). At the time of writing, this variant was classified as a Variant of Uncertain Significance (VUS) in ClinVar (https://www.ncbi.nlm.nih.gov/clinvar/, accessed 12 August 2023) with no conflicts. This variant was not observed in the general population (gnomAD v2.1.1) and was located at the 3′-end of exon 6 with a SpliceAI delta score of 0.90. RT-PCR revealed allele-specific expression (ASE), represented as the loss of the mutant allele, presumably caused by aberrant splicing that leads to frameshift or nonsense changes and subsequent highly efficient NMD (Figure 1A). The larger product size of the affected allele observed in the EP band suggested that the type of aberrant splicing was a partial inclusion of intron 6. Additionally, an partially exonized intronic sequence (c.642 + 1_642 + 132ins) with a low allele fraction was observed (Figure 1B). This exonization is expected to cause premature termination and subsequent NMD because the included sequence contains a stop codon (TAG; c.642 + 67_642 + 69). Although these findings were consistent with the loss of mutant alleles caused by NMD, to rule out the possibility of allele dropout in Sanger RNA sequencing, RNA primer binding sites were investigated from the DNA sequencing data, and no polymorphisms were observed. Based on the PVS1 evidence added from the nullifying effect observed, the variant was classified as an LPV.

The second variant was a missense variant of *MLH1* (NM_000249.4:c.791A>T, p.His264Leu) detected in two patients with early onset colon cancer (Cases No. 2 and 2-1, Supplementary Table S2). Similar to the variant from Case No. 1, this variant was also positioned at the exon-intron boundary (5′-end of exon 10), with a SpliceAI delta score of 0.17. This variant was absent from the general population and was classified as a VUS in ClinVar at the time of writing. Both patients showed a loss of MLH1 expression based on immunohistochemistry (IHC) of the tissue specimens. RT-PCR and subsequent RNA sequencing revealed exon 10 skipping in both control and patient groups (Case No. 2, Figures 1D, E). However, while the variant allele frequency (VAF) of exon 10 skipped traces was approximately 50% in patients, suggesting aberrant splicing from one allele, that in the control was much lower than 50%, suggesting naturally occurring aberrant splicing of a minor proportion of the allele. This difference was also evident from electrophoresis, in which the intensity of the ∼200 bp band was stronger in the patient sample than in the control (Figure 1D). Technical replicate was performed using different set of primer in this case, and the results were reproducible (Supplementary Table S3; Supplementary Figure S1). Because exon 10 skipping (r.791_884del) leads to a frameshift and loss-of-function, we applied PVS1 to this variant and classified it as LPV. A genetic workup was performed on the family members of Case No. 2, and the results are shown along with their pedigree (Supplementary Figure S2A).

In case 3, the intronic variant of *ATM* (NM_000051.4:c.6096-14A>G, p.?) was observed in a patient with breast cancer having family history of breast cancer in mother and maternal aunt. Notably, this variant was classified as ‘Likely Benign (LBV)’ in ClinVar by multiple (5) submitters without conflict in interpretation. However, this variant was predicted to affect splicing (SpliceAI delta score 0.84), and RNA sequencing revealed exon 42 skipping (r.6096_6198del), resulting in a frameshift and premature termination (Figures 1G, H). Therefore, this variant was reclassified as an LPV.

In case 4, the intronic variant of *STK11* (NM_000455.5:c.734 + 5G>A, p.?) was observed in a patient who was clinically compatible with Peutz-Jeghers syndrome. According to medical records, the patient’s father and younger brother showed similar symptoms. This variant was predicted to affect splicing (SpliceAI delta score 0.47), and RNA sequencing showed a partial deletion of exon 5 (r.706_734del), which was also predicted to cause premature termination by a frameshift (Figures 2A, B). Although this variant is classified as Pathogenic in ClinVar, the exact changes in its RNA expression have not been described. Combined with the segregation that had been observed in a family of five members (Mehenni et al., 1998), this variant was classified as a PV.

In case 5, a canonical splice site variant of *BRCA1* (NM_007294.4:c.670 + 1G>A, p.?) were observed in a patient who underwent a medical checkup and had a family history of cancer. Notably, this variant was classified as VUS in ClinVar because of the naturally occurring skipping of exons 8 and 9, an in-frame deletion event. RT-PCR electrophoresis showed a smaller band in both the control and the patient, presumably due to a naturally occurring exon-skipping event (Figure 2D). However, another band was observed, and subsequent RNA sequencing revealed exon 9 skipping, an out-of-frame deletion that caused NMD (Figure 2E). Since exon 9 skipping (r.594_670del) leads to a frameshift and loss-of-function, we applied PVS1 to this variant and classified it as LPV.

In case 6, an intronic INDEL variant involving the canonical splice site (NM_003073.5:c.986 + 1_986 + 10delinsTTGGGTTAA, p.?) was detected in *SMARCB1*. The spliceAI delta score was 0.49 and RT-PCR electrophoresis showed a smaller band (Figure 2G), and RNA sequencing showed exon 7 skipping (r.796_986del; Figure 2H). This variant was observed in a 22-month-old female patient with an atypical teratoid rhabdoid tumor. This variant has not previously been reported in ClinVar. Genetic workup was performed on the parents, and the results are shown along with their pedigree (Supplementary Figure S2B).

In-frame insertions of intronic sequences were observed in *BRCA1/2* genes from three patients with breast or ovarian cancer (Cases 7, 8, and 9). All these 3 variants showed similar predictions from SpliceAI, with loss of the original splice site; however, RNA analysis revealed the creation of a new splice site, resulting in the creation of a longer exon length due to the inclusion of intronic sequences.

Case 7 involved a patient with bilateral breast cancer and a family history of a younger sister and daughter. RNA analysis of the VUS in *BRCA1* (NM_007294.4:c.4186-11C>A, p.?) showed a 9 bp insertion of an intronic sequence (c.4186-9_4186-1ins, Supplementary Figures S3A, B). In this patient, the causative PV was found in *BRCA2* (NM_000059.4:c.7480C>T, p.Arg2494*, data not shown).

Case 8 involved a patient with ovarian cancer and a family history of pancreatic cancer (elder sister) and gastric cancer (elder brother). RNA analysis of the VUS in *BRCA1* (NM_007294.4:c.5407-11T>A, p.?) showed a 9 bp insertion of an intronic sequence (c.5407-9_5407-1ins, Supplementary Figures S3D, E).

Case 9 involved a patient with breast cancer who showed triple-negative IHC of tissue specimens. The patient had no family history of breast or ovarian cancer. An RNA study of the VUS in *BRCA2* (NM_000059.4:c.8755-19A>G, p.?) showed an 18 bp insertion of an intronic sequence (c.8755-18_8755-1ins, Supplementary Figures S3G, H). In this patient, the causative PV was identified in *BRCA1* (NM_007294.4:c.5496_5506delinsA, p.Val1833Serfs*7; data not shown).

Case 10 involved a patient with a mixed germ cell tumor and no family history of cancer. A missense variant was noted in *MSH3* (NM_002439.5:c.2433A>G, p.Leu811 = ), located near the exon-intron boundary (3′-end of exon 17). RNA sequencing was performed because aberrant splicing could not be ruled out (SpliceAI delta score: 0.17). Aberrant splicing was not detected by RNA sequencing and false negativity caused by NMD was ruled out based on the heterozygous SNV observed in the cDNA (Supplementary Figures S4A, B).

Case No. 11 was a patient with endometrial cancer and a history of cancer in the mother (endometrium and colon) and the mother (endometrium). Intronic variants were also observed in *MSH2* (NM_000251.3:c.2635-24A>G). And a spliceAI delta score of 0.69. IHC results for MSH2 in tissue specimens were not available, and an electropherogram of cDNA showed no abnormal findings (Supplementary Figures S4D, E). However, this variant has been listed in ClinVar as a PV/LPV variant by multiple submitters. In a previous study that utilized a next-generation sequencing-based method with NMD inhibition, this variant was found to destroy splicing branch points, activating multiple cryptic splice sites in *MSH2* intron 15, leading to multiple unstable transcripts (Casadei et al., 2019). Considering that these unstable transcripts had a low abundance and that the majority of them might have resulted in NMD, it was not feasible to detect the exact effect of this variant by simple Sanger RNA sequencing without NMD inhibition.

## Discussion

Despite its essential role in interpreting potential splice variants, the clinical use of RNA sequence analysis remains limited. In addition to specimen accessibility and stability, additional costs and turnaround times are obstacles to the widespread application of confirmatory RNA sequence analysis of variants found in DNA sequencing. In this study, we described the results and interpretation procedure of 11 cases of Sanger RNA sequencing, including six cases that escalated from VUS to LPV. Considering that some of these variants are repeatedly found at our institute, suggesting allele frequencies higher than those reported in public databases, the results described in this study could be useful references for other clinicians or geneticists who encounter the same variants in the future. A description of how we interpreted the Sanger RNA sequencing results under the effect of NMD could also provide insights for other laboratories.

To overcome the effect of NMD, Sanger sequencing of RNA should be performed with NMD inhibitor which requires the process of short-term culture of fibroblasts or mononuclear cells (Whiley et al., 2014; Anna and Monika, 2018). However, considering that the limit of detection of Sanger sequencing is approximately 15% (Tsiatis et al., 2010), if the amount of remnant transcripts is higher than this, aberrant splicing can still be detected, regardless of NMD. There has been a misunderstanding that NMD is an all-or-none phenomenon as determined by the canonical rules of NMD. Even in the ACMG guidelines (Richards et al., 2015), it is stated that variants predicted to induce NMD lead to complete absence of gene product. As mentioned in the Introduction, recent studies have shown that this assumption is frequently invalid (Lindeboom et al., 2019; Supek et al., 2021). NMD efficiency is a continuous function with a wide distribution determined not only by canonical rules but also by various parameters, including genetic and non-genetic parameters, and presumably those not yet discovered (Gardner, 2010; Lindeboom et al., 2016; Nomakuchi et al., 2016; Hoek et al., 2019; Karousis and Muhlemann, 2019; Lindeboom et al., 2019; Mauger et al., 2019; Supek et al., 2021). According to the results of this study, only a small fraction of variants expected to undergo NMD seem to result in almost complete mRNA degradation to a level that is not detectable by Sanger RNA sequencing. This high diagnostic yield might have resulted from the incomplete NMD efficiency in blood cells. In one study, blood specimens showed reduced NMD efficiency than fibroblasts resulting in the detection of variants only from blood specimens (Magyar et al., 2009). A number of reports have discussed the variability in NMD efficiency among different tissue types and cell types (Linde et al., 2007; Magyar et al., 2009; Sato and Singer, 2021; Teran et al., 2021) and only one report claimed stable NMD efficiency among tissue types (Teran et al., 2021). Future study on this topic could help specimen selection in RNA sequence analysis.

ASE can be utilized as a surrogate for the degree of NMD for the RNA sequence analysis of an exonic variant, as in Case No. 1 in this study. Distinction between allele dropout and loss of heterozygosity caused by NMD can be achieved by ruling out allele dropout by investigating RNA primer binding sites, as DNA sequencing data are available in the scenario of confirmatory RNA sequencing. More importantly, the primer design process can utilize this information to prevent allele dropout. For confirmatory tests for intronic variants, which constitute the majority of cases in the real world, exonic heterozygous variants can be searched and included as targets of RNA sequence analysis, if present, for ASE-based interpretation of NMD.

As in this study, there do exist small proportion of cases in which the Sanger RNA sequencing results cannot be interpreted because of NMD. Considering its cost-effectiveness, Sanger RNA sequencing without NMD inhibition could be performed as a screening test for aberrant splicing identification, after which technically more demanding tests can be performed in the case of indeterminate results, as suggested by (Bournazos et al., 2022).

In conclusion, the effect of potential splicing variants was identified in 10 of the 11 variants of tumor suppressor genes by Sanger sequencing of RNA without NMD inhibition. The results described in this study could serve as a useful reference for other clinicians or geneticists who encounter the same variants in the future. Sanger RNA sequencing without NMD inhibition is valuable in terms of cost-effectiveness in clinical settings.

## Data Availability

The datasets presented in this study can be found in online repositories. The names of the repository/repositories and accession number(s) can be found in the article/Supplementary Material.
