# Supplementary figures and images for: Reclassification of variants of tumor suppressor genes based on Sanger RNA sequencing without NMD inhibition
Source: Front Genet. 2023 Oct 12;14:1283611. doi: 10.3389/fgene.2023.1283611 (PMC10602670; doi:10.3389/fgene.2023.1283611)

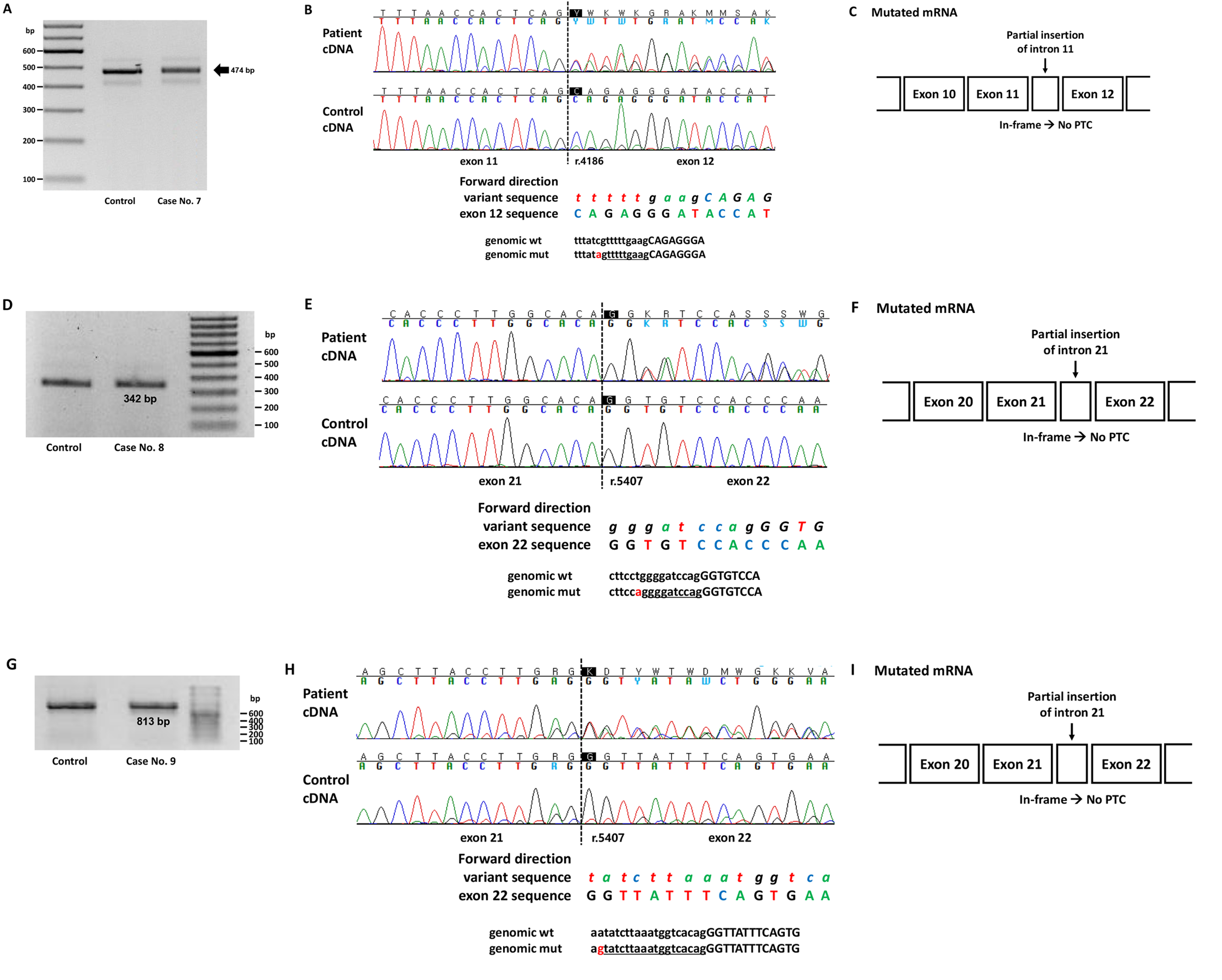

Supplement: Supplementary file 1 [file Image3.TIF]

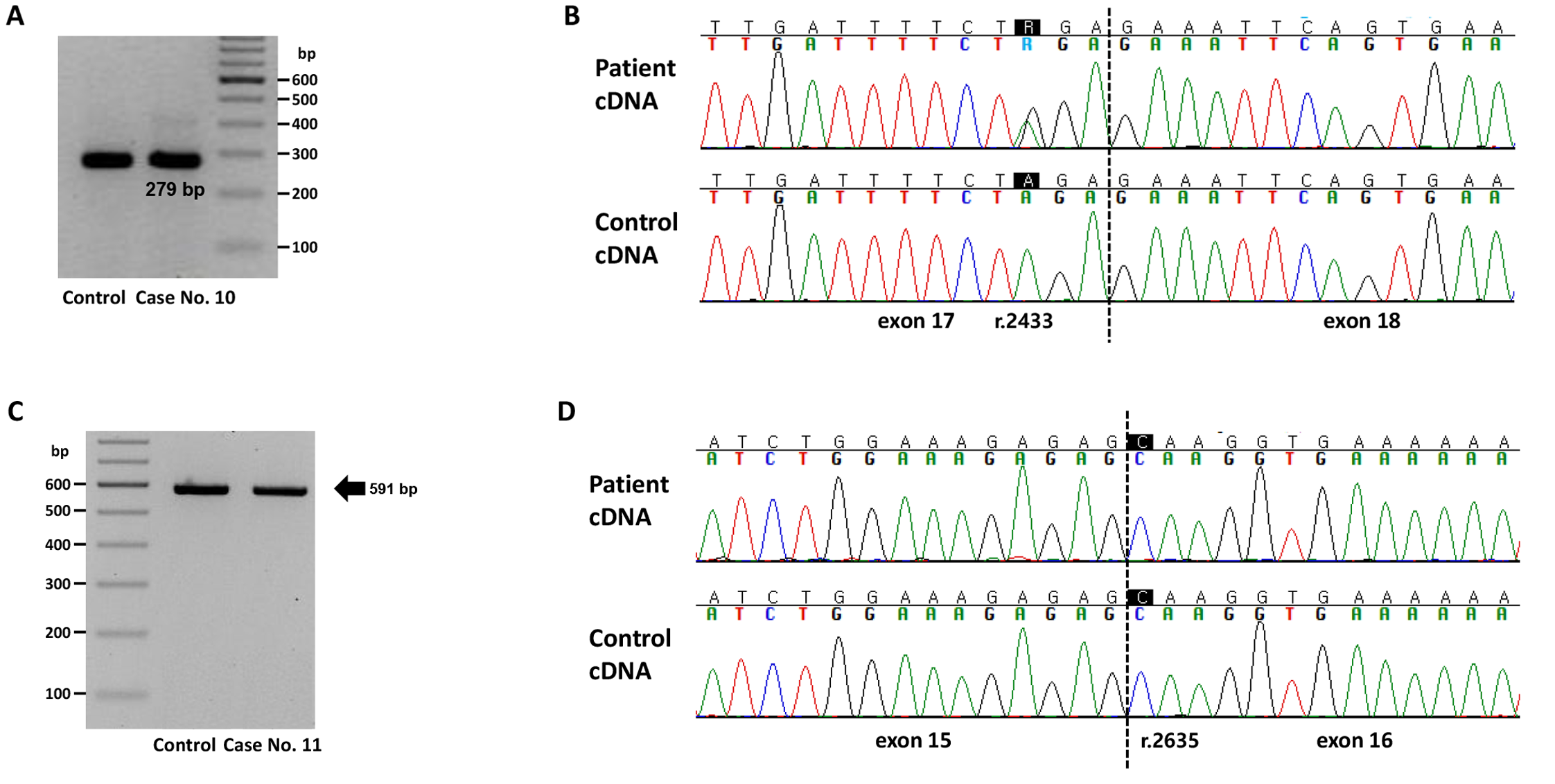

Supplement: Supplementary file 2 [file Image4.TIF]

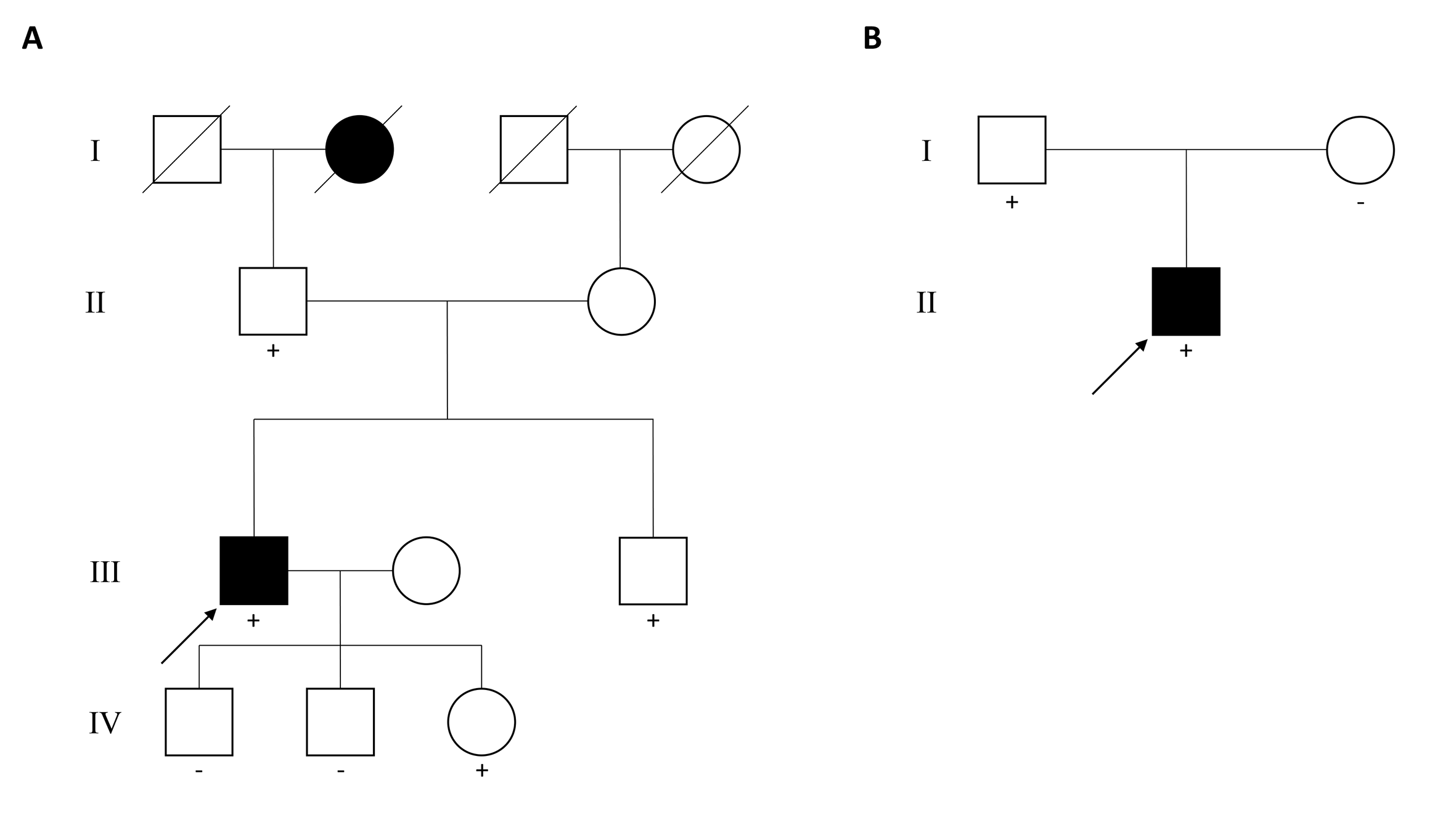

Supplement: Supplementary file 3 [file Image2.TIF]

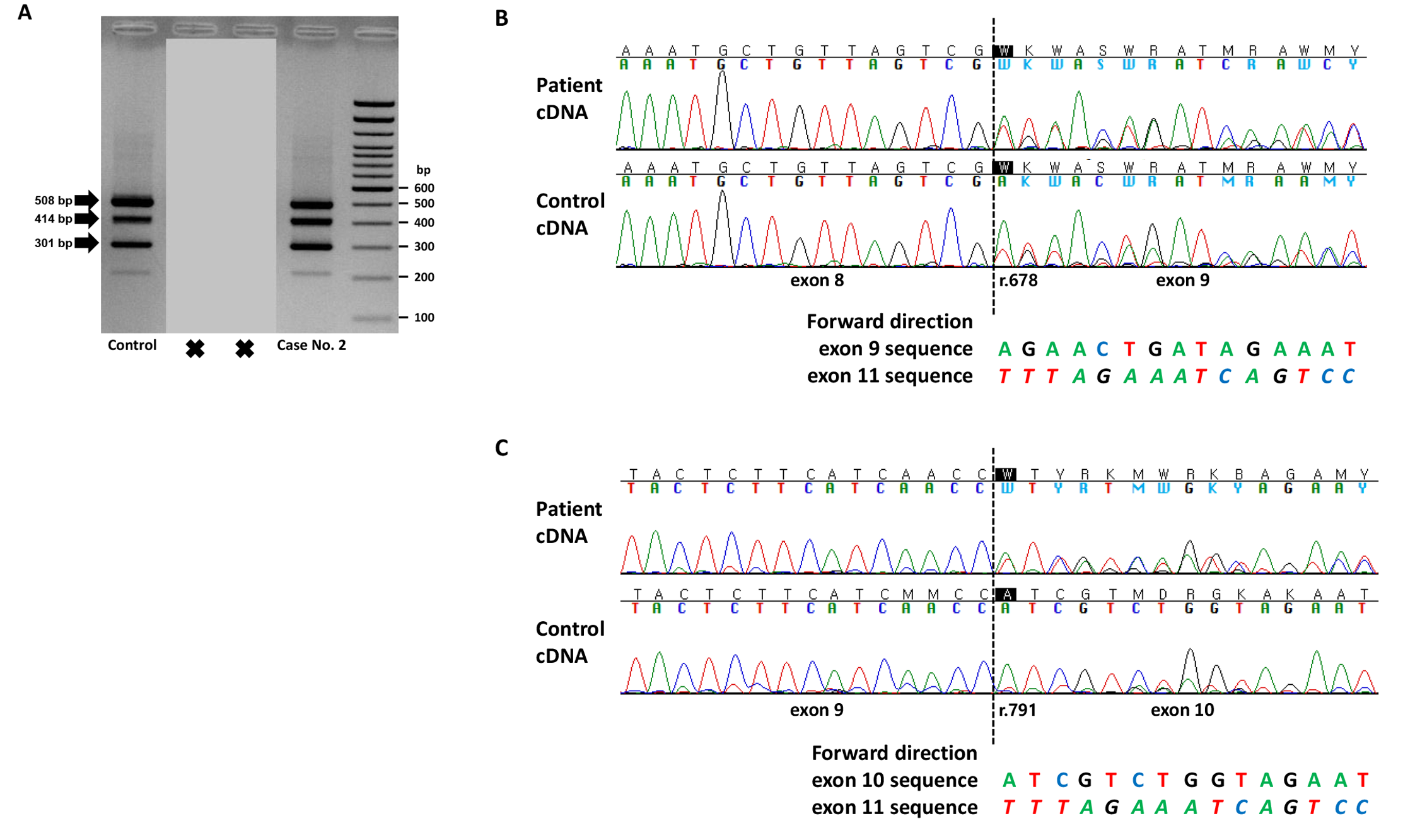

Supplement: Supplementary file 4 [file Image1.TIF]
