# Supplementary material for: Reclassification of variants of tumor suppressor genes based on Sanger RNA sequencing without NMD inhibition
Source: Front Genet. 2023 Oct 12;14:1283611. doi: 10.3389/fgene.2023.1283611 (PMC10602670; doi:10.3389/fgene.2023.1283611)
Supplement: Supplementary file 5 [file DataSheet1.docx]

Supplementary Material

# Supplementary Figures

**Supplementary Figure S1.** Technical duplicate study result of case No. 2 (NM_000249.4(MLH1):c.791A>T). (A) RT-PCR electrophoresis result showing increased allele-specific expression of the mutant allele in case No. 2. (B) cDNA sequencing result showing skipping of exons 9 and 10, with difference in peak height between case No. 2 (50%) and control (<50%). In the cDNA sequences, partial 5’-end of exon 11 are italicized. (C) cDNA sequencing result showing identical result with FIGURE 1D, a skipping of exon 10, with difference in peak height between case No. 2 (50%) and control (<50%). In the cDNA sequences, partial 5’-end of exon 11 are italicized.

**Supplementary Figure S2.** Pedigrees of the families from cases (A) No. 2 (NM_000249.3(MLH1):c.791A>T) and (B) No. 6 (NM_003073.3(SMARCB1):c.986+1_986+10delinsTTGGGTTAA). An arrow indicates the proband, a square indicates male, a circle indicates female, a filled symbol indicates affected, and crossed line through a symbol indicates the individual is deceased. Individuals who have done genetic test, their result is descripted below a square or circle, either “+” (variant detected) or “-” (variant not detected).

**Supplementary Figure S3.** Genetic study result of cases No. 7 (NM_007294.4(BRCA1):c.4186-11C>A), No. 8 (NM_007294.4(BRCA1):c.5407-11T>A) and No. 9 (NM_000059.4(BRCA2):c.8755-19A>G). (A) RT-PCR electrophoresis result of case No. 7. (B) cDNA sequencing result showing 9 bp insertion of intronic sequence in case No. 7. (C) Diagram of the mutated mRNA of case No. 7. Since this variant causes in-frame insertion, premature termination codon was not existed. (D) RT-PCR electrophoresis result of case No. 8. (E) cDNA sequencing result showing 9 bp insertion of intronic sequence in case No. 8. (F) Diagram of the mutated mRNA of case No. 8. Since this variant causes in-frame insertion, premature termination codon was not existed. (G) RT-PCR electrophoresis result of case No. 9. (H) cDNA sequencing result showing 18 bp insertion of intronic sequence in case No. 9. (I) Diagram of the mutated mRNA of case No. 9. Since this variant causes in-frame insertion, premature termination codon was not existed. (B), (E), (H) Below, the sequences of wild type and mutated genomic DNA are presented. Nucleotide substitution is in red, and the nucleotides inserted in the cDNA are underlined. Small letters indicate intronic sequence. (C), (F), (I) Box sizes are not proportional to the length of exon and intron.

**Supplementary Figure S4.** Genetic study result of cases No. 10 (NM_002439.5(MSH3):c.2433A>G) and No. 11 (NM_000251.3(MSH2):c.2635-24A>G). (A) RT-PCR electrophoresis result of case No. 10. (B) cDNA sequencing result showing no aberrant splicing in case No. 10. (C) RT-PCR electrophoresis result of case No. 11. (D) cDNA sequencing result showing no aberrant splicing in case No. 11.
